# Beyond Healthiness: The Impact of Traffic Light Labels on Taste Expectations and Purchase Intentions

**DOI:** 10.3390/foods9020134

**Published:** 2020-01-28

**Authors:** Sonja Kunz, Simona Haasova, Jannik Rieß, Arnd Florack

**Affiliations:** Department of Psychology, University of Vienna, 1010 Vienna, Austria; simona.haasova@univie.ac.at (S.H.); jannik.riess@factum.at (J.R.); arnd.florack@univie.ac.at (A.F.)

**Keywords:** traffic light labels, nutrition labels, healthiness, tastiness, purchase intention, food products, sugar

## Abstract

The aim of traffic light labels on food products is to help consumers assess their healthiness. However, it is not clear whether traffic light labels do not have undesired side effects by signaling lower tastiness of healthy product alternatives and reducing purchase intentions. We therefore conducted a study with consumers from Austria (*N* = 173) in which we presented the amount of sugar contained in products on labels with or without traffic light colors based on the coding criteria of the UK Food Standards Agency. Expectations of products’ healthiness and tastiness, as well as purchase intentions were assessed. The products were randomly sampled from the category of desserts from a supermarket. The declared amount of sugar was experimentally varied. The traffic light labels helped participants differentiate between the healthiness of products with different sugar levels. They did not affect the expected tastiness of the healthier alternatives. Moreover, participants did not report lower purchase intentions for products high in sugar, but a higher purchase intention for products low in sugar when traffic light colors were used compared to when they were not used.

## 1. Introduction

A balanced, healthful diet is one of the most important factors that contributes to staying healthy and avoiding all kinds of diseases. Yet, 39% of adults of the world population are overweight or obese and thus more at risk for developing cardiovascular diseases, diabetes, and certain types of cancer [1]. One of the major causes for overweight and obesity is an increased consumption of high-energy foods, which contain a lot of fat or sugar. Thus, preventing overconsumption of sugary and fatty foods is substantial to facilitate healthier nutrition throughout the population. Accordingly, various health campaigns label processed food products in a way that allows consumers to assess important dietary information about the products. One popular approach, already practiced in the UK, is to place *traffic light nutrition labels* on food items. The traffic light labels indicate the levels of four key nutrients (i.e., fat, sugar, saturates, and salt) commonly contained in processed food, with red indicating a high level, amber a medium level, and green a low level of the respective nutrient. The purpose of the traffic light system is to advise consumers against choosing products with a high amount of these ingredients and thus assisting them in making healthier food choices [2]. However, research findings are inconclusive regarding the system’s effectiveness [3,4,5,6,7,8,9].

In particular, it is unclear whether usage of traffic light labeling might increase the attractiveness of unhealthy food as an undesired side effect. Indeed, two related lines of reasoning suggest that such undesired effects could occur. First, traffic light labels might elicit reactance, an unpleasant motivational state that people experience when they feel threatened in their personal freedom of choice and which renders the “forbidden”, in this case unhealthy, alternative more attractive [10]. Second, traffic light labels might shift consumers’ focus on products’ healthiness and activate an unhealthy = tasty intuition [11] according to which consumers expect unhealthy alternatives to taste better than the healthy ones.

In the present study, we therefore examined the effects of labels with traffic light colors indicating the amount of sugar in a food product on participants’ healthiness and tastiness ratings as well as purchase intentions of this product. Furthermore, we examined whether such traffic light labeling on food products leads consumers to experience a threat to freedom of their choice and whether their expectations of the food’s healthiness and tastiness fall in line with the unhealthy = tasty intuition.

### 1.1. Theoretical Background

Traffic light nutrition labels have been introduced as a simple way of indicating the healthiness of a food product, aiming to help consumers make healthier food choices [2]. Research findings suggest that traffic light nutrition labels indeed improve peoples’ accuracy in estimation of foods’ healthiness [12,13,14]. However, findings on the effectiveness of traffic light nutrition labels in promoting healthy eating are mixed. Whereas some studies suggest that traffic light labels can encourage healthier eating behavior [3,4,5], other studies did not find any effects of traffic light labels on sales or consumption of healthy food [6,7,8,9].

Whereas the conditions under which desired, undesired, and no effects of traffic light nutrition labels occur are not completely clear, research in social psychology and marketing provides possible explanations for undesired effects of such labels. Traffic light labels might be viewed as a threat to freedom, eliciting reactance and thus decreasing attractiveness of “promoted” healthy products. Moreover, traffic light labels might shift consumers’ focus specifically on the health aspect of products. Ironically, the focus on healthiness together with the perception of restricted freedom of choice might give way to the belief that healthy eating can only be achieved with effort and thus comes with the cost of sacrificing the food’s taste [11]. In this light, the ease with which healthy products can be identified by the traffic light labels [12,13,14] might make these products appear less tasty and less desirable.

#### 1.1.1. Reactance as a Driver of Undesired Effects of Nutrition Labels

A theoretical explanation for the possible negative effects of traffic light labels on products’ desirability draws on research on the theory of reactance [10]. People are in a state of psychological reactance when they feel that their personal freedom is threatened or eliminated [10]. As a result, people feel the need to restore their personal freedom and the “forbidden” object or behavior becomes more attractive [15]. People can either restore their freedom directly by obtaining the denied object, or indirectly, by subjectively increasing the attractiveness of the denied option, while decreasing the attractiveness of possible alternatives [16]. Anything that hinders people in their free behavior constitutes a possible threat to freedom.

In the domain of consumer research, different factors such as promotional influence, product unavailability, and pricing [15] have been found to elicit reactance. When a product is imposed on consumers, they may favor it less than when they are not pressured to buy it [17]. Contrastingly, when people had to choose between different products and were informed that one of the products was unexpectedly unavailable, people’s liking for the unavailable product increased [18]. Increased liking of denied and decreased liking of imposed options has been observed in many consumer-related domains [15]. Particularly relevant for the present research, there are plenty of cases in which health promotions have been ineffective or led to the opposite of the intended effects [19,20,21,22]. For example, explicit compared to implicit anti-smoking messages have been shown to increase intentions to smoke in 10th graders [23]. A qualitative approach surveying college students found that anti-smoking campaigns are perceived as a threat but fail to actually help smokers quit and instead might evoke annoyance and defiance [24]. Similar results were found for messages intended to prevent people from alcohol consumption [25,26,27] and drug use [28,29].

In the domain of healthy eating, research showed that the prohibition of snacks can increase children’s consumption of these snacks [30,31]. Similarly for adults, a negative description of unhealthy food or explicit health messages have been found to increase dieters’ desire and consumption of unhealthy food [32,33,34]. However, only few studies directly examined reactance elicited by nutrition labels. One exception is a study by Wagner, Howland, and Mann [35]. In this study, participants were less likely to choose a healthy product when it was labeled with an explicit health message, compared to a subtle message. In another study [36], participants rated how much they wanted to taste full-, reduced-, and no-fat cream cheese. The cream cheese was presented either with an explicit health warning label, an information label displaying the percentage of fat, or without any label. Participants in the warning label condition, compared to the information label condition, indicated an increased desire to taste the full-fat cheese.

#### 1.1.2. Beliefs about the Negative Relation between Health and Taste as a Driver for Undesired Effects

The reactance theory implies that the perception of a threat to freedom of choice leads to a motivation to restore this freedom, most likely by choosing the restricted alternative. Hence, a traffic light label “warning” the consumer against a product by indicating low healthiness (usually with red color) might increase the consumer’s motivation to choose it. Additionally, the need to apply such labels to a product might already suggest that the respective product must be attractive because otherwise no label would be necessary to prevent a consumer from choosing it. Hence, traffic light labels might activate the belief that unhealthy products taste good. The idea that higher level goals such as a healthy diet cannot be reached with enjoyment and ease but require sacrifices is the essence of the Protestant work ethic [37] and, according to Raghunathan et al. [11], a source for the unhealthy = tasty intuition. Use of the traffic light system on food labeling might highlight exactly these qualities associated with the healthy eating goal, thus eliciting negative taste expectations towards healthy food and positive taste expectations towards unhealthy food. Moreover, as taste is an important driver of purchase intention [38], this might also lead to lower intentions to purchase healthy products and higher intentions to purchase unhealthy products.

#### 1.1.3. Alternative Theoretical Accounts

Despite the extensive line of argumentation emphasizing the possible pitfalls of traffic light labels, there are also some theoretical accounts implying that health labels do not necessarily affect taste expectations in a negative way. Haasova and Florack [39], for example, argue that consumers rely on nutritional information when forming health judgments, but that it is more difficult for consumers to translate this information into taste expectations. They reason that taste judgments are based on mental simulation more than health judgments. Moreover, there is evidence that consumers form health and taste judgments based on similar cues and often in a congruent way [40], implying that the two types of judgments can be positively related. Furthermore, some findings suggest that reactance does not necessarily impact behavior in a negative way. For example, Cho et al. [41] found that reactance to anti-smoking messages does not interfere with quitting. In a study by Wang et al. [42], health labels affected healthiness but not tastiness judgments. Similarly, Bullock et al. [43] found that a health warning message affected the perceived healthiness of ice cream but not their overall liking.

### 1.2. Hypotheses

While informational nutrition labels leave room for interpretation about the healthfulness of a product, research showed that traffic light colored labels unambiguously indicate a product’s healthiness and improve peoples’ accuracy of estimating the healthiness of food products [12,13,14]. Therefore, we hypothesized that traffic light labels on food products influence the perceived healthiness of products, such that differences in expected healthiness between products containing a low, medium, and high amount of sugar increase when sugar labels are presented in traffic light colors.

In addition, reasoning based on the reactance theory [10] and research on the perceived relation between healthiness and tastiness [11] leads to the hypotheses that the application of traffic light colors leads to higher taste expectations and purchase intentions for unhealthier products (i.e., high in sugar) and lower taste expectations and purchase intentions for healthier products (i.e., low in sugar). Based on this line of research, we also assumed that the application of traffic light labels evokes a perceived threat to freedom of choice for unhealthier products. Furthermore, as traffic light labels might activate an unhealthy = tasty intuition, we expected that participants indicate a stronger belief that unhealthy products taste better than healthy products after being exposed to products with traffic light labels compared to being exposed to products without traffic light labels. For the same reason, we hypothesized that the correlation between healthiness and tastiness expectations is less positive (or more negative) when participants were exposed to products with traffic light labels rather than without traffic light labels.

However, even if these hypotheses are straight forward and based on prominent lines of research, it has to be noted that some of the studies we reviewed above indicated that health-related information is not always linked to negative taste expectations [39,42,43] and health judgments are often positively correlated with taste expectations [40,44]. It is therefore important to shed light on the possible impact of traffic light labels on healthiness and tastiness expectations and the related impact on purchase intentions.

## 2. Method

We conducted an online study with a sample of Austrian consumers and presented participants pictures of unknown products from the category “desserts” (e.g., puddings, mousses, and rice puddings). We used desserts as stimuli because the sugar content is highly relevant in this category and varies substantially between products. We varied the products’ sugar level (low vs. medium vs. high) and whether the sugar level was presented on labels with or without traffic light colors (traffic light labels vs. neutral labels).

In order to increase the ecological validity of the study, we used a large set of products retrieved from an online shop of a foreign supermarket and randomly sampled products for each participant out of the complete product pool. We used unknown products to avoid the possibility that any knowledge about the actual sugar content of the presented products could interfere with the manipulation of the displayed sugar content. In the condition with traffic light labels indicating the amount of sugar, we applied the coding criteria prescribed by the UK Food Standards Agency [2]. We measured the healthiness and tastiness expectations and purchase intentions for the presented products. For each participant, 20 pictures of products were randomly drawn from a pool of 213 pictures, then presented and rated one after another. Furthermore, we assessed whether participants perceived a threat to freedom of choice for each product. In addition, we assessed for each participant to what extent she or he explicitly believed in the unhealthy = tasty intuition [11]. Finally, we also controlled for individual characteristics such as body mass index (BMI), trait reactance [45], and general health interest [46].

### 2.1. Participants

A sample of consumers from Austria (selected to match the distribution of age, gender, and educational background of the Austrian population) recruited through the “Talk Online Panel” [47] participated in the online study. The Talk Online Panel Ltd. is a continuous panel, for which people can sign up to participate in surveys in exchange for rewards. A total of 173 consumers participated in the survey in exchange for 2.50 € per participant, of which 16 participants were excluded because their healthiness and tastiness ratings showed no variance and had a standard deviation of 0, indicating that they merely “clicked through” the questionnaire. The final sample then consisted of 157 participants (see Table 1 for descriptive statistics of the sample).

### 2.2. Materials

In the present study, the stimulus material originated from a pool of 71 pictures of food products from the category “desserts” available in the online store of the Belgian supermarket Delhaize, at the time of the study. Products included puddings, rice puddings, semolina puddings, flans, mousses, and dessert creams with different flavors (mostly chocolate, vanilla, coffee, and caramel), as well as popular desserts such as crème brûlée or tiramisu. A nutrition label was added to each picture, displaying the amount of sugar per 100 g of each dessert. Three different versions of every picture were created, varying in the displayed amount of sugar on the nutrition label (*low, medium, high*). The medium version displayed the actual amount of sugar per 100 g, as indicated on the supermarket’s online shop. In order to create products varying more strongly in the displayed amount of sugar, we decreased the actual amount of sugar by 75% for the low sugar version and increased the amount of sugar by 50% for the high sugar version. The displayed amount of sugar varied randomly between 1.05 and 40.2 g per 100 g. In total, there was a pool of 213 pictures with varying sugar levels for each condition. Each participant saw 20 pictures, drawn randomly from this stimulus pool.

**Figure 1:**
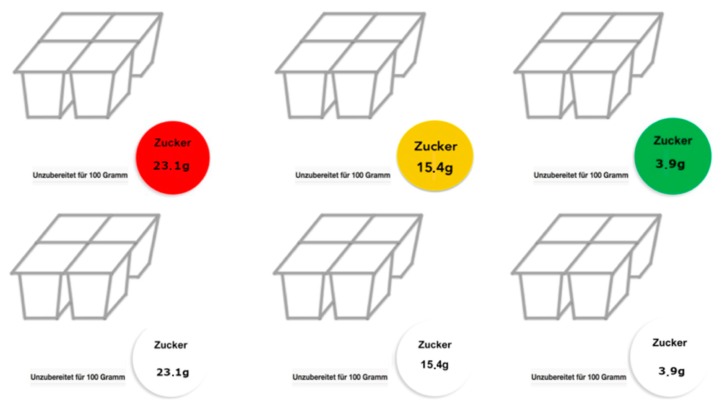
Example of stimulus material used in the traffic light label (upper three icons) and the neutral label (lower three icons) condition. In the study, all products were presented on separate screens. Please note that, due to copyright reasons, here we used a graphical icon for the sake of illustration.

In the traffic light label condition, the labels were colored according to the color coding prescribed by the UK Food Standards Agency [2]: If the displayed amount of sugar per 100 g was 5 g or lower, the label was colored green. If the amount of sugar was larger than 5 g and lower than or equal to 22.5 g, the label was colored amber. If the amount of sugar was higher than 22.5 g per 100 g, the label was colored red. In the neutral label condition, all three versions were colored white. For an illustrative example of the stimulus material, see Figure 1.

### 2.3. Design and Procedure

**Figure 2:**
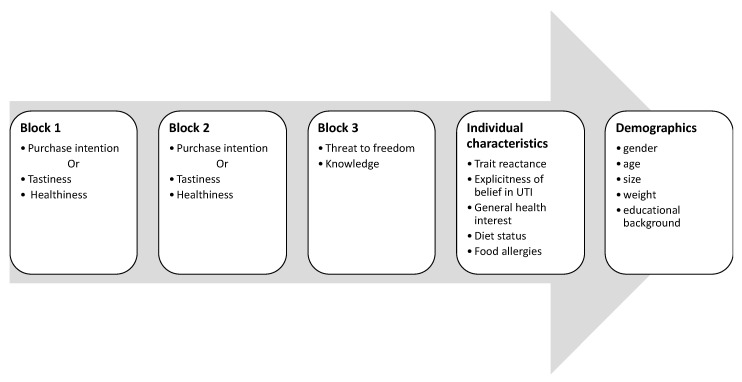
Overview of the experimental procedure. Note that each of the first three blocks consisted of 20 stimulus pictures, presented and rated one after another in random order. The procedure was the same for participants in both conditions.

The study employed a three (sugar level: High, medium, low) × two (label condition: Traffic light label vs. neutral label) mixed design, with sugar level varying within and label condition between participants. Participants were randomly assigned to one of two label conditions (*traffic light label* vs. *neutral label*). Participants in the traffic light label condition were introduced to the concept of traffic light nutrition labels and explained the meaning of the different label colors. The further experimental procedure was identical for participants in both conditions (see Figure 2 for an overview of the procedure). The questionnaire and an English translation can be found in the Supplementary Material.

Participants were asked to rate the perceived tastiness and healthiness of 20 product pictures in one block. They viewed and immediately rated each product before moving on to the next. Participants rated how tasty they estimated the presented products would be on a horizontal 11-point rating scale, with response options ranging from 1 (*not at all tasty*) to 11 (*very tasty*), allowing for a neutral answer. We assessed the healthiness expectations with an 11-point staple scale format: “How healthy do you estimate the presented product to be?” with response options ranging from +5 to +1 (indicating healthiness) and −1 to −5 (indicating unhealthiness), displayed vertically underneath each other without option labels. Participants were told, “+5 indicates a very high estimate of a product’s healthiness”, and “−5 indicates a very low estimate of a product’s healthiness.” The scale included the response option 0 in between the two staples, which was labelled with “neither healthy nor unhealthy”. For the statistical analyses, the healthiness scores were subsequently recoded to correspond to the tastiness scores (*1 = very unhealthy* and *11 = very healthy*). By employing two different scale formats to measure the two constructs of healthiness and tastiness, we avoided the “common scale format” as a source of potential common method variance [48], which, due to the correlational and proximate nature of our measurements, could possibly inflate the relationships found between the measured variables. In a second block, we assessed participants’ purchase intention for each product. Participants indicated how likely they would buy each product, on an 11-point horizontal scale ranging from 1 (*not at all likely*) to 11 (*very likely*). The order of the two rating blocks, one for tastiness and healthiness, another for purchase intention, as well as of the pictures in each block was randomized.

In a third block, we assessed whether traffic light labels constituted a *threat to freedom* with a single-item measure. For each product picture, participants indicated their agreement to the statement “Do you feel restricted in your free choice?” (1 = *not at all*, 10 = *a lot*; [49]). To assess participants’ *knowledge* about the meaning of the nutrition labels (i.e., recommended amount of intake by nutrition experts), in the same sequence, all participants also indicated their agreement to the statement “Nutrition experts would recommend a limited intake of this product” (1 = *I do not agree*, 10 = *I agree a lot*).

After administering the product ratings, we asked participants for demographics: Age, gender, size, and weight to compute their BMI and educational background. Moreover, we asked whether they were currently on a diet and whether they had any food allergies. Eventually, we assessed individual characteristics. We measured the explicitness of belief in the unhealthy = tasty intuition by asking for agreement on a 9-point rating scale (1 = *strongly disagree*; 9 = *strongly agree*) to the items: (1) *“Things that are good for me rarely taste good”* and (2) *“There is no way to make food healthier without sacrificing the taste”* [11]. We assessed participants’ trait reactance using five items of Hong’s psychological reactance scale [45] with a 5-point scale from 1 = *strongly disagree* to 5 = *strongly agree*: (1) *“I understand advice from others to be an intrusion”,* (2) *“I become frustrated when I am unable to make free and independent choices”*, (3) “*Advice and recommendations usually induce me to do just the opposite*”, (4) *“I find contradicting others stimulating”*, and (5) “*When something is prohibited, I usually think ‘that’s exactly what I am going to do’*”. In addition, we measured participants’ general health interest with eight items from Roininen et al. [46] on a 7-point scale from 1 = *strongly disagree* to 7 = *strongly agree* (e.g., *“I always follow a healthy and balanced diet”*). For descriptive statistics of these variables and the scales’ Cronbach’s alpha coefficients, see Table 2.

### 2.4. Data Analysis

We used linear mixed-effect model analyses [50] to test our hypotheses. We conducted all analyses with the SPSS statistics software (Version 25). For the linear mixed-effect model analyses, all continuous variables were centered on their grand means. All models included a random intercept per participant, meaning that the model intercept was allowed to vary freely between participants. Parameters were estimated using maximum likelihood. The significance level was *α* = 0.05 for all analyses. In all linear mixed-effect model analyses, we also ran additional models in which we controlled for the individual differences in BMI, trait reactance, and general health interest. Controlling for these factors did not change the significance value nor the direction of any of the results.

## 3. Results

### 3.1. Effects of Traffic Light Labels on Knowledge and Perceived Threat to Freedom

#### 3.1.1. Knowledge about Recommendations

To examine whether the traffic light labels support consumers in interpreting the sugar content, we ran a linear mixed-effect model with *knowledge* as an outcome variable and label condition (traffic light label vs. neutral label), sugar level (low, medium, high), and their two-way interaction as predictors. The model results are depicted in Table 3. Across conditions, participants expected more strongly that nutrition experts would recommend a limited intake of products with a high amount of sugar than for products with a low amount of sugar, *F*(2, 2998.44) = 332.94, *p* < 0.001. However, the significant interaction between condition and sugar level, *F*(2, 2998.44) = 4.86, *p* = 0.01, indicates that this differentiation was moderately stronger when traffic light labels were used, compared to the condition without traffic light labels (for estimated marginal means and contrast tests, see Table 4).

#### 3.1.2. Threat to Freedom

To examine if traffic light labels constituted a threat to freedom for products with a higher amount of sugar, we again conducted a linear mixed-effect model analysis, predicting *threat to freedom* from the label condition (traffic light label vs. neutral label), the sugar level (low, medium, high), and their two-way interaction. The results of the model test are depicted in Table 3. The interaction of sugar level and label condition on the perceived *threat to freedom* was significant, *F*(2, 2989.30) = 5.65, *p* = 0.004 (for estimated marginal means and contrast tests, see Table 4). Whereas the perceived *threat to freedom* was higher for both the original and high sugar level compared to the low sugar level in the traffic light label condition, in the condition without traffic light labels, it was only higher in the low compared to the high sugar level. Notably, the perceived *threat to freedom* was generally quite low in all conditions (all means < 3 on a scale from 1 to 10).

### 3.2. Effects of Traffic Light Labels on Expected Healthiness, Expected Tastiness, and Purchase Intentions

#### 3.2.1. Influence of Traffic Light Labels on Healthiness Expectations

To test whether traffic light labels help assess product healthiness, based on sugar level, we ran a linear mixed-effect model, with healthiness as a dependent variable, while label condition, sugar level, and their two-way interaction remained the predictors (see Table 5 for all model results). In line with the hypotheses, there was a significant interaction between label condition and sugar level, *F*(2, 2999.23) = 18.87, *p* < 0.001 (for estimated marginal means and contrast tests, see Table 6). In both conditions, perceived healthiness was higher, the lower the products’ sugar level. However, the differences in perceived healthiness between the sugar levels were more pronounced when traffic light labels were present compared to when they were not present.

#### 3.2.2. Influence of Traffic Light Labels on Taste Expectations

To test whether traffic light labels compared to neutral labels affect the perceived tastiness of low, medium, and high sugar products, we conducted another linear mixed-effect model with label condition, sugar level, and their interactions as predictors, whereas tastiness was the dependent variable (see Table 5 for all model results). We hypothesized that higher sugar levels are associated with increased tastiness expectations in the condition with traffic light labels compared to the condition without traffic light labels, whereas a low sugar level is associated with decreased tastiness in the condition with traffic light labels compared to the condition without traffic light labels. However, the interaction between sugar level and label condition was not significant, *F*(2, 3006.01) = 1.04, *p* = 0.36. Yet, there was a significant main effect of sugar level on perceived tastiness, *F*(2, 3006.01) = 3.37, *p* = 0.04, indicating that participants expected products with a low sugar level to be slightly tastier compared to the products with a high level of sugar (for estimated marginal means and contrast tests, see Table 6).

#### 3.2.3. Influence of Traffic Light Labels on Purchase Intentions

To test whether traffic light labels affect the purchase intention of products labeled with a low, medium, and high sugar level, we conducted a linear mixed-effect model, predicting purchase intention from label condition, sugar level, and their interaction (see Table 5 for all model results). There was a significant interaction between label condition and sugar level, *F*(2, 3005.07) = 7.45, *p* = 0.001 (for estimated marginal means and contrast tests, see Table 7). However, the effect was not in line with our hypothesis. There was no significant difference in purchase intentions between the label conditions for products with a high sugar level, and participants indicated higher purchase intentions for products with a low sugar level in the condition with traffic light labels compared to the condition without traffic light labels.

### 3.3. Effects of Traffic Light Labels on Associations between Healthiness and Tastiness

We expected that the traffic light system would strengthen the belief that unhealthy food tastes better. Hence, we tested effects of the use of the traffic light system on the strength of participants’ explicit belief in the unhealthy = tasty intuition and the individual level correlation between healthiness and tastiness, as well as between healthiness and purchase intentions.

#### 3.3.1. Influence of Traffic Light Labels on the Belief that Unhealthy Food Tastes better than Healthy Food

To test whether traffic light labels activate the belief that unhealthy food tastes better (unhealthy = tasty intuition), we conducted an independent samples *t*-test of participants’ explicitness of belief in the unhealthy = tasty intuition between the traffic light label and the neutral label condition. The difference between the condition with traffic light labels and the condition without traffic light labels was not significant, *t*(155) = −0.29, *p* = 0.78. Hence, we found no evidence that the presence of traffic light labels influenced participants’ strength of the explicit belief that unhealthy food tastes better than healthy food.

#### 3.3.2. Influence of Traffic Light Labels on the Healthiness–Tastiness Relationship

To test whether traffic light labels affect healthiness–tastiness associations at the level of individual food items, we ran a linear mixed-effect model, with tastiness as the dependent variable and the label condition, healthiness, and their two-way interaction as predictors (see Table 8 for all model results). In contrast to the hypothesis, the interaction between label condition and healthiness was not significant, *b* = −0.07 (*SE* = 0.04), *t*(3132.19) = −1.64, *p* = 0.10. Yet, there was a significant main effect of healthiness on tastiness, *b* = 0.17 (*SE* = 0.03), *t*(3109.14) = 5.10, *p* < 0.001, corroborating a weak, but significant positive healthiness–tastiness association. However, this effect was not moderated by the label condition, suggesting that traffic light labels did not influence the healthiness–tastiness relationship.

#### 3.3.3. Influence of Traffic Light Labels on the Healthiness–Purchase Intention Relationship

To test whether traffic light labels affect purchase intentions for subjectively healthy vs. unhealthy food, we adapted the linear mixed-effect model used above, exchanging tastiness with purchase intention as an outcome variable while healthiness, label condition, and their two-way interaction remained the predictors (see Table 8 for all model results). There was no significant interaction between label condition and healthiness, *b* = −0.02 (*SE* = 0.05), *t*(3116.32) = −0.34, *p* = 0.73. There was a significant main effect of healthiness on purchase intention, *b* = 0.34 (SE = 0.04), *t*(3076.78) = 9.41, *p* < 0.001, indicating a positive relationship between subjective product healthiness and purchase intention. This effect was not moderated by the label condition, indicating that traffic light labels do not affect the purchase intentions for subjectively healthy and unhealthy products.

## 4. Discussion

Currently, traffic light labels are employed as a tool for consumers to visually recognize foods’ healthiness status and thus foster their healthy food choices. Since different countries consider making traffic light labels on food products obligatory, it is crucial to investigate their effects on food expectations and evaluations, specifically their possible negative effects that might arise due to reactance elicited by the labeling system. Whereas traffic light labels have been shown to improve the accuracy of healthiness estimations of food products [12,13,14], it has been unknown whether they have the unwanted side effect of reducing perceived tastiness and purchase intentions of food products considered healthier, such as products low in sugar. To investigate whether traffic light labels affect tastiness expectations and purchase intentions of products in an undesired way, we presented participants with dessert products varying in the amount of sugar that was displayed. The label displaying the amount of sugar was either white or colored according to the traffic light coding system.

We found that traffic light labels help participants differentiate products’ healthiness based on their numerical sugar content. Our findings indicate that consumers already use numerical information about the sugar content to differentiate between product healthiness, as has been demonstrated in prior research [39]. Yet, traffic light labels still increased differences in healthiness ratings between the different sugar levels. Our findings are in line with previous research, demonstrating improved healthiness estimations through traffic light labels [13,14,51]. Possibly, colored traffic light labels indicate products’ healthiness more clearly than the mere numerical information. They may facilitate translating the amount of sugar into the healthiness estimation of a product [51,52] by signaling explicitly and straightforwardly which products are considered healthy and unhealthy, respectively.

Contrary to our expectations, traffic light labels did not affect the products’ expected tastiness nor purchase intentions in a negative way. In particular, neither expected tastiness nor purchase intentions for low sugar products decreased when traffic light labels were present. In fact, purchase intentions for low sugar products increased slightly when traffic light labels were present. A potential explanation for this finding is that participants might have answered in a socially desirable manner. As they were told that traffic light labels are meant to encourage healthy food choices, they might have accordingly indicated higher purchase intentions for low sugar products with a green label. However, the presence of traffic light labels did not reduce purchase intentions for high sugar products, indicating that social desirability did not apply to these responses and thus did not represent a general issue in our study. Prior research already suggested that consumers use the numerical sugar value of products as an indicator for healthiness, but not for tastiness [39]. Similarly, it was found that green colored nutrition labels, as currently used in Nordic countries, increased the healthiness perceptions of snacks without changing tastiness perceptions [42]. Our study adds to these findings, by demonstrating that traffic light labels indicating the amount of sugar on a product affect healthiness expectations but not tastiness expectations of products.

In the same vein, we found no evidence for a reactance effect towards traffic light labels. The perceived threat to freedom of traffic light labels was in fact very low and possibly could not have led to a reactance effect. Moreover, traffic light labels did not affect the unhealthy = tasty intuition. Participant’s explicit belief in the unhealthy = tasty intuition did not change when traffic light labels were present. Similarly, associations between healthiness and tastiness of individual products were unaffected by traffic light labels. Since there is no reactance effect of traffic light labels, an important question is who benefits most from such labels. We did not find that the traffic light labels reduced the purchase intentions for desserts high in sugar. However, it is possible that individuals who are effective in self-regulation [53] or those with a strong prevention focus [54,55,56] use the information provided by traffic light labels and avoid products high in sugar or consume less of them. Hence, future research on the effect of traffic light labels on purchase intentions and consumption behavior might study individual level moderators such as consumers’ regulatory focus as well.

An important limitation of our study is that the labels we used exclusively referred to the amount of sugar per 100 g, whereas we did not present amounts of other nutrients (e.g., fat, salt, carbohydrates) that are usually presented together in the traffic light labeling system. Moreover, the labels were not directly attached to the product but presented next to it. Therefore, participants might have used the nutrition labels in this study differently than they would in a real-life setting. Future research may use a combination of differently colored labels displaying the amounts of different nutrients and place them on the actual product, in order to create a more naturalistic study context. Furthermore, we assessed the products’ expected tastiness with rating scales and participants did not have a chance to really taste any of the products. Therefore, our finding that traffic light labels do not affect product tastiness is restricted to subjective expected tastiness based on viewing pictures of products. Our results are still relevant because consumers in real life often have to evaluate products merely based on their appearance and usually do not have the opportunity to taste them before purchasing, for example when shopping for products in a supermarket. However, food labels have been shown to impact taste perception as well [57]. It would be an important goal for future research to test whether traffic light labels affect the real tasting experience of products. Similarly, we assessed purchase intentions merely with rating scales. Future studies might test the effect of traffic light labels on real purchase behavior.

In summary, our research implies that traffic light labeling used to indicate the amount of sugar could help consumers differentiate between healthy and less healthy food products. At the same time, these labels do not seem to have any negative effects on the expected tastiness and purchase intentions of products. However, our study does not indicate that traffic light colors reduce the purchase intentions for products high in sugar either. We conclude that the application of visual labels according to the traffic light system might be used as a tool to assist consumers in making healthier food choices without taxing products’ desirability.

## Figures and Tables

**Table 1:** Descriptive sample statistics in separate conditions.

	Overall (N = 157)	Traffic Light Label (N = 79)	Neutral Label (N = 78)
*Age (years)*			
Mean (SD)	49.20 (18.01)	49.49 (17.57)	48.91 (18.56)
Minimum-Maximum	18–83	20–83	18–80
*Gender*			
Male	82 (52.2%)	47 (59.5%)	35 (44.9%)
Female	75 (47.8%)	32 (40.5%)	43 (55.1%)
*BMI*			
Mean (SD)	25.52 (4.26)	25.28 (4.07)	25.77 (4.46)
Minimum-Maximum	16.60–38.51	16.60–37.45	18.37–38.51
*Diet*			
Yes	6 (3.4%)	5 (6.3%)	1 (1.3%)
No	151 (96.2%)	74 (93.7%)	77 (98.7%)
*Food allergies*			
Yes	26 (16.6%)	14 (17.7%)	12 (15.4%)
No	131 (83.4%)	65 (82.3%)	66 (84.6%)
*Educational background*			
Compulsory school	31 (19.7%)	20 (25.3%)	11 (14.1%)
Middle/secondary school	4 (2.5%)	4 (5.1%)	-
Vocational school/training	79 (50.3%)	40 (50.5%)	39 (50.0%)
High school	24 (15.3%)	7 (8.9%)	17 (21.8%)
University/college degree	18 (11.5%)	8 (10.1%)	10 (12.8%)
Other	1 (0.6%)	-	1 (1.3%)

*Note.* SD: Standard deviation; the distributions of the quantitative variables age and BMI are described in terms of their mean (SD) and range. The distributions of the categorical variables diet status, food allergies, gender, and educational background are described in terms of frequencies (%).

**Table 2:** Descriptive statistics and Cronbach’s alpha coefficients of the measured scales.

Variable	*M (SD)*	*α*
Belief in unhealthy = tasty intuition	4.17 (2.27)	0.81
General health interest	4.31 (1.09)	0.81
Trait reactance	2.45 (0.75)	0.70

*Note.* M: Mean; SD: Standard deviation.

**Table 3:** Parameter estimates of the effects of sugar level and condition on knowledge and threat to freedom, using linear mixed-effect model analysis.

Parameter	Outcome Variable	
	Knowledge	Threat to Freedom
*Fixed effects*		
Intercept	8.16 *** (0.26)	2.69 *** (0.23)
Condition	0.27 (0.36)	0.02 (0.32)
Sugar level	−2.09 *** (0.13)	−0.21 ** (0.08)
Condition × Sugar level	−0.59 ** (0.19)	−0.31 ** (0.11)

*Note.* Values are parameter estimates predicting the evaluations of products. Standard errors appear in parentheses. Condition is a dichotomous variable coded as follows: 0 = traffic light label condition, 1 = neutral label condition. Sugar level is a categorical variable coded as follows: 0 = “low sugar”, 1 = “original sugar”; 2 = “high sugar”. ** *p* < 0.01. *** *p* < 0.001.

**Table 4:** Estimated marginal means of knowledge and threat to freedom ratings for products with low, original, and high sugar level in the traffic light label and neutral label condition.

	Knowledge				Threat to Freedom		
	*M*				*M*		
	*Overall*	Traffic Light Label	Neutral Label		*Overall*	Traffic Light Label	Neutral Label
*Overall*		7.22 _x_	7.26 _x_	*Overall*		2.50 _x_	2.59 _x_
Low sugar	5.90 _a_	5.74 _ax_	6.07 _ax_	Low sugar	2.33 _a_	2.18 _ax_	2.47 _ax_
Original sugar	7.53 _b_	7.50 _bx_	7.56 _bx_	Original sugar	2.61 _b_	2.63 _bx_	2.60 _abx_
High sugar	8.29 _c_	8.43 _cx_	8.16 _cx_	High sugar	2.70 _b_	2.71 _bx_	2.69 _bx_

*Note.* M = mean. Means with different subscripts a,b,c between rows and x,y between columns are significantly different at *p* < 0.05 in paired contrasts.

**Table 5:** Parameter estimates of the effects of sugar level and condition on healthiness evaluations, tastiness evaluations, and purchase intentions, using linear mixed-effect model analysis.

Parameter	Outcome Variable		
	Healthiness	Tastiness	Purchase Intention
*Fixed effects*			
Intercept	3.53 *** (0.19)	5.90 *** (0.22)	3.64 *** (0.24)
Condition	−0.34 (0.27)	0.09 (0.31)	0.24 (0.34)
Sugar level	1.31 *** (0.10)	0.13 (0.14)	0.73 *** (0.06)
Condition × Sugar level	0.86 *** (0.14)	0.23 (0.20)	0.73 ** (0.21)

*Note.* Values are parameter estimates predicting the evaluations of products. Standard errors appear in parentheses. The continuous variables in the model are centered on a grand mean. Condition is a dichotomous variable coded as follows: 0 = traffic light label condition, 1 = neutral label condition. Sugar level is a categorical variable coded as follows: 0 = “low sugar”, 1 = “original sugar”; 2 = “high sugar”. ** *p* < 0.01. *** *p* < 0.001.

**Table 6:** Estimated marginal means of healthiness and tastiness ratings for products with low, original, and high sugar level in the traffic light label and neutral label condition.

	Healthiness				Tastiness		
	*M* (*SD*)				*M* (*SD*)		
	*Overall*	Traffic Light Label	Neutral Label		*Overall*	Traffic Light Label	Neutral Label
*Overall*		4.20 _x_	4.06 _x_	*Overall*		6.13 _x_	5.97 _x_
Low sugar	5.10 _a_	5.35 _ax_	4.84 _ax_	Low sugar	6.19 _a_	6.35 _ax_	6.0 _ax_
Original sugar	3.96 _b_	4.08 _bx_	3.82 _bx_	Original sugar	6.00 _ab_	6.03 _abx_	5.96 _ax_
High sugar	3.35 _c_	3.18 _cx_	3.53 _cx_	High sugar	5.95 _b_	6.00 _bx_	5.90 _ax_

*Note.* M = mean. Means with different subscripts a,b,c between rows and x,y between columns are significantly different at *p* < 0.05 in paired contrasts.

**Table 7:** Estimated marginal means of purchase intentions for products with low, original, and high sugar level in the traffic light label and neutral label condition.

	Purchase Intention		
	*M (SD)*		
	*Overall*	Traffic Light Label	Neutral Label
*Overall*		4.51 _x_	4.01 _x_
Low sugar	4.85 _a_	5.33 _ax_	4.37 _ay_
Original sugar	4.16 _b_	4.31 _bx_	4.02 _ax_
High sugar	3.76 _c_	3.88 _cx_	3.64 _bx_

*Note.* M = mean. Means with different subscripts a,b,c between rows and x,y between columns are significantly different at *p* < 0.05 in paired contrasts.

**Table 8:** Parameter estimates of the effects of healthiness and condition on tastiness expectations and purchase intentions, using linear mixed-effect model analysis.

Parameter	Outcome Variable	
	Tastiness	Purchase Intention
*Fixed effects*		
Intercept	5.98 *** (0.21)	4.03 *** (0.21)
Condition	0.14 (0.29)	0.45 (0.29)
Healthiness	0.17 *** (0.03)	0.34 *** (0.04)
Condition × Healthiness	−0.07 (0.04)	−0.02 (0.05)

*Note.* Values are parameter estimates predicting the evaluations of products. Standard errors appear in parentheses. The continuous variables in the model are centered on grand mean. Condition is a dichotomous variable coded as follows: 0 = traffic light label condition, 1 = neutral label condition. *** *p* < 0.001.

## Supplementary Materials

The questionnaire used in this study, along with an English translation, can be found in the online version of this paper. Research data to this article, as well, can be found online at https://www.mdpi.com/2304-8158/9/2/134/s1.
