# Supplementary material for: Beyond Healthiness: The Impact of Traffic Light Labels on Taste Expectations and Purchase Intentions
Source: Foods. 2020 Jan 28;9(2):134. doi: 10.3390/foods9020134 (PMC7074546; doi:10.3390/foods9020134)
Supplement: Supplementary file 1 [file foods-09-00134-s001.zip › Supplementary files/Questionnaire_german.pdf]

Fragebogen

1 Informed Consent

Liebe Teilnehmerin, lieber Teilnehmer,

herzlichen Dank für Ihre Bereitschaft, an dieser Befragung teilzunehmen. In dieser Studie untersuchen wir Konsumverhalten im Ernährungsbereich.

Die Befragung wird etwa 15 Minuten in Anspruch nehmen.

Die Studie dient ausschließlich wissenschaftlichen Zwecken. Die Befragung wird vom Institut für Angewandte Psychologie: Arbeit, Bildung, Wirtschaft der Universität Wien durchgeführt. Alle Informationen, die wir von Ihnen erhalten, werden vertraulich behandelt und anonymisiert ausgewertet, sodass keine Rückschlüsse auf Ihre Person möglich sind.

- Im Rahmen der Studie werden Ihre Daten kodiert, d.h. im Falle der Abfrage von persönlichen Daten (z.B. Name; IP-Adresse; etc.), werden diese strikt von den Untersuchungsdaten (z.B. Fragebogendaten) getrennt. Durch diese Kodierung wird im Rahmen der wissenschaftlichen Auswertung kein Unbefugter Ihre persönlichen Daten erhalten. Nach Beendigung der Untersuchung werden alle Daten gelöscht, die einen Bezug zu Ihrer Person erlauben. Die Daten werden nicht an Personen weitergegeben, die an der Studie nicht beteiligt sind. In eine mögliche Veröffentlichung der Resultate der Untersuchung gehen die Daten anonymisiert ein. Wir sichern Ihnen zu, dass alle von uns erhobenen Daten entsprechend dem Datenschutzgesetz geschützt werden.

Mit dem Klicken des “Weiter”-Buttons, bestätigen Sie, die Einleitung gelesen zu haben, und willigen ein, an dieser Studie teilzunehmen:

"Ich bin damit einverstanden, dass meine Angaben ausschließlich für wissenschaftliche Zwecke am Institut für Angewandte Psychologie: Arbeit, Bildung, Wirtschaft aufbewahrt und ausgewertet werden. Nach Beendigung des Forschungsvorhabens werden alle Daten gelöscht, die einen Bezug zu meiner Person erlauben."

Bei Fragen, wenden Sie sich bitte an sonja.kunz@univie.ac.at

Vielen Dank für Ihre Teilnahme an der Studie!

2.1 Introduction Traffic Light Labels

Here you fill in your question text.

Here you explain how the question has to be filled in (optional).

Sie werden im Verlauf dieses Fragebogens Bilder von Lebensmitteln zu sehen bekommen. Es gilt diese nach verschiedenen Kriterien zu bewerten. Die Lebensmittel sind dabei mit Lebensmittelampeln gekennzeichnet.

Lebensmittelampeln sind Kennzeichnungen auf Lebensmitteln, die darstellen sollen, wie gesund ein Produkt ist. Um ungesunder Ernährung in der Gesellschaft vorzubeugen, wird in immer mehr Ländern die Einführung von einfach verständlichen Kennzeichnungen von Lebensmitteln gefordert.

In unserem Beispiel zeigen Ampeln den Zuckergehalt des Produktes an. Grün steht dabei für einen niedrigen Gehalt von Zucker, Gelb für einen mittleren Anteil und Rot für einen hohen Anteil von Zucker.

Lebensmittelampeln sollen bewirken, dass die Bevölkerung immer mehr auf ungesunde Produkte verzichtet.

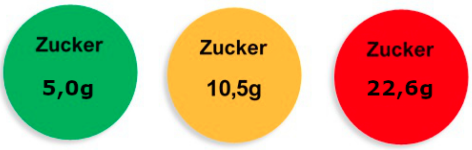

2.2.1 Purchase Intention

Wir zeigen Ihnen nun 20 einzelne Produkte. Geben Sie bitte für jedes Produkt an, wie wahrscheinlich Sie das Produkt kaufen würden. Es ist klar, dass Sie viele Produkte nicht kennen oder noch nie konsumiert haben. Uns geht es nur um Ihre Schätzung. Gehen Sie nach dem Eindruck, den das jeweilige Produkt auf Sie in einem Supermarkt machen würde.

Bitte beurteilen Sie jedes Produkt einzeln. Ihre Beurteilung ist für uns sehr wichtig.

2.2.1.1.1 Purchase Intention

Wie wahrscheinich würden Sie dieses Produkt kaufen?

- ○ Gar nicht wahrscheinlich
- ○
- ○
- ○
- ○

- ○
- ○
- ○
- ○
- ○
- ○ Sehr wahrscheinlich

2.2.2 Healthy-Tasty

Wir zeigen Ihnen nun 20 einzelne Produkte. Beurteilen Sie jedes Produkt danach, wie lecker und wie gesund es Ihrer Meinung nach ist. Es ist klar, dass Sie viele Produkte nicht kennen oder noch nie konsumiert haben. Uns geht es nur um Ihre Schätzung. Gehen Sie nach dem Eindruck, den das jeweilige Produkt auf Sie in einem Supermarkt machen würde.

Bitte beurteilen Sie jedes Produkt einzeln. Ihre Beurteilung ist für uns sehr wichtig.

2.2.2.1.1 Healthy-Tasty

Wie lecker empfinden Sie das Produkt?

- ○ Überhaupt nicht lecker
- ○
- ○
- ○
- ○
- ○
- ○
- ○
- ○
- ○
- ○ Sehr lecker

Wie gesund schätzen Sie das Produkt ein?

Je weiter oben Sie auf der Skala ankreuzen desto gesünder beurteilen Sie das Produkt.

+ 5 bedeutet eine sehr hohe Einschätzung der Gesundheit
– 5 bedeutet eine sehr niedrige Einschätzung der Gesundheit

- ○ +5
- ○ +4
- ○ +3
- ○ +2
- ○ +1
- ○ 0 - weder gesund noch ungesund
- ○ -1
- ○ -2
- ○ -3
- ○ -4
- ○ -5

2.3 Reaktanz

Wir zeigen Ihnen nun 20 einzelne Produkte und fragen Sie nach Ihrem Konsumverhalten. Es ist klar, dass Sie viele Produkte nicht kennen oder noch nie konsumiert haben. Uns geht es nur um Ihre Schätzung. Gehen Sie nach dem Eindruck, den das jeweilige Produkt auf Sie in einem Supermarkt machen würde.

Bitte beurteilen Sie jedes Produkt einzeln. Ihre Beurteilung ist für uns sehr wichtig.

2.3.1.1 Reaktanz

Fühlen Sie sich in Ihrer Entscheidungsfreiheit eingeschränkt?

- ○ Gar nicht
- ○
- ○
- ○
- ○
- ○
- ○
- ○
- ○
- ○
- ○ Sehr

Aus Sicht von Ernährungsexpertinnen und -experten sollte man sich beim Konsum dieses Produktes stark einschränken.

- ○ Stimme nicht zu
- ○
- ○
- ○
- ○
- ○
- ○
- ○
- ○
- ○
- ○ Stimme sehr zu

3.1 LUA_control

Here you fill in your question text.

Here you explain how the question has to be filled in (optional).

Sie werden im Verlauf dieses Fragebogens Bilder von Lebensmitteln zu sehen bekommen. Es gilt diese nach verschiedenen Kriterien zu bewerten. Es geht dabei nicht um richtige oder falsche Antworten, sondern um Ihre persönliche Einschätzung.

3.2.1 Purchase Intention

Wir zeigen Ihnen nun 20 einzelne Produkte. Geben Sie bitte für jedes Produkt an, wie wahrscheinlich Sie das Produkt kaufen würden. Es ist klar, dass Sie viele Produkte nicht kennen oder noch nie konsumiert haben. Uns geht es nur um Ihre Schätzung. Gehen Sie nach dem Eindruck, den das jeweilige Produkt auf Sie in einem Supermarkt machen würde.

Bitte beurteilen Sie jedes Produkt einzeln. Ihre Beurteilung ist für uns sehr wichtig.

3.2.1.1.1 Purchase Intention

Wie wahrscheinich würden Sie dieses Produkt kaufen?

- ○ Gar nicht wahrscheinlich
- ○
- ○
- ○
- ○
- ○
- ○
- ○
- ○
- ○
- ○ Sehr wahrscheinlich

3.2.2 Healthy-Tasty

Wir zeigen Ihnen nun 20 einzelne Produkte. Beurteilen Sie jedes Produkt danach, wie lecker und wie gesund es Ihrer Meinung nach ist. Es ist klar, dass Sie viele Produkte nicht kennen oder noch nie konsumiert haben. Uns geht es nur um Ihre Schätzung. Gehen Sie nach dem Eindruck, den das jeweilige Produkt auf Sie in einem Supermarkt machen würde.

Bitte beurteilen Sie jedes Produkt einzeln. Ihre Beurteilung ist für uns sehr wichtig.

3.2.2.1.1 Healthy-Tasty

Wie lecker empfinden Sie das Produkt?

- ○ Überhaupt nicht lecker
- ○
- ○
- ○
- ○
- ○
- ○
- ○
- ○
- ○
- ○ Sehr lecker

Wie gesund schätzen Sie das Produkt ein?

Je weiter oben Sie auf der Skala ankreuzen desto gesünder beurteilen Sie das Produkt.

+ 5 bedeutet eine sehr hohe Einschätzung der Gesundheit
– 5 bedeutet eine sehr niedrige Einschätzung der Gesundheit

○ +5

○ +4

○ +3

○ +2

○ +1

○ 0 - weder gesund noch ungesund

○ -1

○ -2

○ -3

○ -4

○ -5

3.3 Reaktanz

Wir zeigen Ihnen nun 20 einzelne Produkte und fragen Sie nach Ihrem Konsumverhalten. Es ist klar, dass Sie viele Produkte nicht kennen oder noch nie konsumiert haben. Uns geht es nur um Ihre Schätzung. Gehen Sie nach dem Eindruck, den das jeweilige Produkt auf Sie in einem Supermarkt machen würde.

Bitte beurteilen Sie jedes Produkt einzeln. Ihre Beurteilung ist für uns sehr wichtig.

3.3.1.1 Reaktanz

Fühlen Sie sich in Ihrer Entscheidungsfreiheit eingeschränkt?

○ Gar nicht

○

○

○

○

○

○

○

○

○

○ Sehr

Aus Sicht von Ernährungsexpertinnen und -experten sollte man sich beim Konsum dieses Produktes stark einschränken.

○ Stimme nicht zu

○

○

○

○

○

- ○
- ○
- ○
- ○
- ○ Stimme sehr zu

4 Info Board Skalen

Vielen Dank für die Beantwortung der Fragen.

Wir werden Ihnen nun noch einige Fragen zu Ihrem Verhalten sowie Ihren persönlichen Einstellungen stellen. Wir bitten Sie, ehrlich und spontan zu antworten. Es geht nur um Ihre Selbsteinschätzung und Ihre Meinung. Es gibt daher keine richtigen oder falschen Antworten.

5 Trait Reactance

Bitte geben Sie an, in welchem Ausmaß die folgenden Aussagen auf Sie zutreffen.

Sie können zwischen "Trifft gar nicht zu" und "Trifft vollständig zu" wählen.

	Trifft gar nicht zu				Trifft vollständig zu
Ich verstehe den Rat von anderen als Einmischung.	○	○	○	○	○
Ich bin frustriert, wenn ich keine freien und unabhängigen Entscheidungen treffen kann.	○	○	○	○	○
Ratschläge und Empfehlungen bringen mich meist dazu, genau das Gegenteil zu machen.	○	○	○	○	○
Ich empfinde es als belebend, anderen zu widersprechen.	○	○	○	○	○
Wenn etwas verboten ist, denke ich mir für Gewöhnlich: "Genau das werde ich tun".	○	○	○	○	○

6 Explicitness of believe

Bitte geben Sie an, wie sehr Sie mit den Aussagen übereinstimmen:

	1 - Stimme überhaupt nicht zu	2	3	4	5	6	7	8	9 - Stimme vollkommen zu
Produkte, die gut für mich sind, schmecken selten gut.	○	○	○	○	○	○	○	○	○
Man kann Speisen und Produkte nicht gesünder machen, ohne dass der Geschmack darunter leidet.	○	○	○	○	○	○	○	○	○

7 general health interest

Bitte geben Sie an, wie sehr Sie mit den Aussagen übereinstimmen:

	1 - Stimme überhaupt nicht zu	2	3	4	5	6	7 - Stimme vollkommen zu
Wie gesund ein Produkt ist, hat keine Auswirkungen auf meine Wahl bei Lebensmitteln.	○	○	○	○	○	○	○
Ich bin sehr genau, wenn es darum geht, wie gesund Nahrungsmittel sind.	○	○	○	○	○	○	○
Ich esse, was ich will, und mache mir keine Gedanken über die Nährwerte des Produkts.	○	○	○	○	○	○	○
Für mich ist es wichtig, dass ich wenig Fett zu mir nehme.	○	○	○	○	○	○	○
Ich achte sehr auf eine ausgewogene und gesunde Ernährung.	○	○	○	○	○	○	○
Ich achte darauf, dass meine tägliche Ernährung reich an Vitaminen und Mineralien ist.	○	○	○	○	○	○	○

Ob ein Snack gesund ist oder nicht macht für mich keinen Unterschied.	○	○	○	○	○	○	○
Ich meide Lebensmittel auch dann nicht, wenn sie eventuell meinen Cholesterinspiegel erhöhen.	○	○	○	○	○	○	○

8 food pleasure orientation

Bitte geben Sie an, wie sehr Sie mit den Aussagen übereinstimmen:

	stimme überhaupt nicht zu	2	3	4	5	6	stimme vollkommen zu
Essen zu genießen, ist eine der größten Freuden in meinem Leben.	○	○	○	○	○	○	○
Ich esse lieber meine Lieblingsspeise, als meine Lieblings-Fernsehsendung zu schauen.	○	○	○	○	○	○	○
Ich denke in freudiger Erwartung an Essen.	○	○	○	○	○	○	○
Geld, das man für Essen ausgibt, ist sinnvoll verwendetes Geld.	○	○	○	○	○	○	○
Ich habe sehr angenehme Erinnerungen an familiäre Anlässe, an denen es Essen gab.	○	○	○	○	○	○	○
Wenn ich meine ernährungsbedingten Bedürfnisse sicher, günstig und ohne Hungergefühl mit der Einnahme einer täglichen Tablette stillen könnte, würde ich das machen.	○	○	○	○	○	○	○

9 Diät

Machen Sie derzeit eine Diät?

- ○ Ja
- ○ Nein

10 Allergien

Haben Sie Lebensmittelunverträglichkeiten und / oder Allergien?

- ○ Ja
- ○ Nein

11 Demographie

Welches Geschlecht haben Sie?

- ○ weiblich
- ○ männlich
- ○ anderes ____

Welche Staatsangehörigkeit haben Sie?

- ☐ Deutsch
- ☐ Österreich
- ☐ Schweiz
- ☐ andere EU
- ☐ Andere Nicht-EU

Ist Deutsch ihre Muttersprache?

- ○ Ja
- ○ Nein

Wenn Deutsch nicht Ihre Muttersprache ist, wie gut sprechen Sie deutsch?

- So gut wie meine Muttersprache
- Sehr gut
- Gut
- Mäßig
- Nicht so gut
- Sehr schlecht

In welchem Jahr sind Sie geboren?

Bitte geben Sie das Jahr in 4 Ziffern an (z.B. 1980).

Geburtsjahr

In welchem Monat sind Sie geboren?

Bitte wählen Sie aus
Januar
Februar
März
April
Mai
Juni
Juli
August
September
Oktober
November
Dezember

Wie groß sind Sie?

Größe in cm

Was ist Ihr Gewicht?

Gewicht in KG

Sind Sie zurzeit erwerbstätig?

Unter Erwerbstätigkeit wird jede bezahlte bzw. mit einem Einkommen verbundene Tätigkeit verstanden, egal welchen zeitlichen Umfang sie hat.

Was auf dieser Liste trifft auf Sie zu?

- Ich bin vollzeit-erwerbstätig mit einer wöchentlichen Arbeitszeit von 35 Stunden und mehr
- Ich bin teilzeit-erwerbstätig mit einer wöchentlichen Arbeitszeit von 15 bis 34 Stunden
- Ich bin teilzeit- oder stundenweise erwerbstätig mit einer wöchentlichen Arbeitszeit unter 15 Stunden
- Ich bin in Mutterschafts-/Erziehungsurlaub oder in sonstiger Beurlaubung
- Ich bin zurzeit nicht erwerbstätig

Wenn Sie nicht vollzeit- oder teilzeiterwerbstätig sind: Zu welcher Gruppe auf dieser Liste gehören Sie?

- Schüler/-in an einer allgemeinbildenden Schule
- Student/-in
- Rentner/-in, Pensionär/-in, im Vorruhestand
- Arbeitslose
- Dauerhaft Erwerbsunfähige
- Hausfrauen/Hausmänner
- Sonstiges

Welchen <u>höchsten</u> allgemeinbildenden Schulabschluss haben Sie?

- Ich bin ohne Abschluss von der Schule abgegangen
- Ich habe einen Abschluss einer Pflichtschule (z.B. Hauptschule oder entsprechende Stufe einer anderen Schulform)
- Ich habe einen Realschulabschluss oder einen vergleichbaren Abschluss
- Ich habe einen Abschluss einer Fachschule oder berufsbildenden Schule
- Ich habe die allgemeine oder fachgebundene Hochschulreife / Abitur / Matura oder die Fachhochschulreife
- Ich habe einen Universitäts- oder Fachhochschulabschluss (Bachelor, Master, Diplom, Magister, Lizenziat, Staatsexamen, Promotion, Habilitation oder andere)
- Ich habe einen anderen Schulabschluss und zwar

12 End

Die Studie ist nun beendet. Vielen Dank für Ihre Teilnahme!

Ziel dieser Studie ist es zu untersuchen, wie Lebensmittelampeln den eingeschätzten Geschmack von Lebensmitteln beeinflussen. Dies ist relevant, da ungesunde Ernährung ein großes Problem auf der gesamten Welt darstellt und zu starken gesundheitlichen Schäden führen kann. Im Moment werden Lebensmittelampeln als eine Möglichkeit diskutiert, eine gesündere Ernährung anzuregen.

Bei der Debatte um das Fördern von gesünderer Ernährung wird allerdings häufig außer Acht gelassen, dass solche Vorstöße auch Reaktanz erzeugen können. Reaktanz entsteht, wenn sich Menschen in ihrer Entscheidungsfreiheit eingeschränkt fühlen. Dies kann sich dann in der Aufwertung von verbotenen Alternativen äußern. Wir denken, dass Lebensmittelampeln die Entscheidungsfreiheit einschränken können. Produkte, die mit roten Ampeln gekennzeichnet werden, werden dabei aufgewertet.

Verschiedene Studien zeigen, dass Menschen normalerweise gesünderes Essen als leckerer einschätzen. Wir erwarten, dass wenn Lebensmittelampeln gezeigt werden, Menschen sich eingeschränkt in Ihrer Wahlfreiheit fühlen und auf einmal die "verbotenen" ungesunden Produkte leckerer bewerten.

Unsere Hypothese ist: Bei Produkten mit Lebensmittelampeln ist der Zusammenhang zwischen eingeschätzter Gesundheit und Schmackhaftigkeit geringer, als bei Produkten ohne Lebensmittelampel.

Dafür bewerten in dieser Studie zwei Gruppen (mit/ohne Lebensmittelampel) Lebensmittel nach ihrer Schmackhaftigkeit und Gesundheit. Außerdem wird überprüft, ob die TeilnehmerInnen sich durch die Produkte in Ihrer Wahlfreiheit eingeschränkt gefühlt haben (Reaktanz). Andere Skalen, wie das generelle Interesse an Gesundheit und ob TeilnehmerInnen gerade auf Diät sind, sollen ausschließen, dass die Ergebnisse durch diese Faktoren verfälscht werden.

Falls Sie weitere Fragen haben oder sich für die Studienergebnisse interessieren, wenden Sie sich bitte an: sonja.kunz@univie.ac.at

Wenn Sie auf 'Weiter' klicken, werden Sie zum Talk Online Panel zurückgeleitet.

Möchten Sie uns noch etwas mitteilen?

Hier können Sie Ihre Gedanken und Eindrücke über die Studie notieren und/oder Feedback zum Fragebogen geben.

13 Endseite
