# Supplementary material for: Beyond Healthiness: The Impact of Traffic Light Labels on Taste Expectations and Purchase Intentions
Source: Foods. 2020 Jan 28;9(2):134. doi: 10.3390/foods9020134 (PMC7074546; doi:10.3390/foods9020134)
Supplement: Supplementary file 1 [file foods-09-00134-s001.zip › Supplementary files/Questionnaire_english.pdf]

Complete questionnaire (study materials in detail)

Note that this is an English translation (approximate; done by the authors) of the questionnaire that was originally provided to the study's participants in German language.

1. Welcome and informed consent

2. Introduction to traffic light labels (traffic light label group only):

   "In this survey, you are going to see pictures of food items. We ask you to evaluate these items according to different criteria. The food items are labelled with traffic light food labels.

   Traffic light labels are labels on food items, which shall indicate how healthy a product is. To prevent unhealthy eating in the society, easily comprehensible labeling of food products is promoted and introduced in many countries.
   In our example, traffic light labels indicate the sugar content of a product. Green is for a low amount of sugar, yellow for a medium amount and red is for a high amount of sugar. The goal of the traffic light labeling system is to help people to refrain from unhealthy food products."

3. Healthiness and tastiness ratings (first block)

Product picture

a. Tastiness estimation

"How tasty do you perceive this product?"

Not at all tasty ○ ○ ○ ○ ○ ○ ○ ○ ○ ○ ○ Very tasty

b. Healthiness estimation

"How healthy do you estimate this product to be?"

"The higher the value on the scale you tick, the healthier you judge the product.

+ 5 means a very high estimate of healthiness
- 5 means a very low estimate of healthiness "

- ○ +5
- ○ +4
- ○ +3
- ○ +2
- ○ +1
- ○ 0 – neither healthy nor unhealthy
- ○ -1
- ○ -2
- ○ -3
- ○ -4
- ○ -5

4. Purchase intention (second block)

Product picture

“How likely would you buy this product?”

Not at all likely										Very likely
○	○	○	○	○	○	○	○	○	○	○

5. Threat to freedom and knowledge ratings (third block)

Product picture

a. Threat to freedom

“Do you feel restricted in your free choice?”

Not at all									A lot
○	○	○	○	○	○	○	○	○	○

b. Knowledge

“Nutrition experts would recommend a limited intake of this product.”

I do not agree									I agree a lot
○	○	○	○	○	○	○	○	○	○

6. Trait reactance

“Please indicate to what extent the following statements apply to you.
You can choose from ‘Does not apply at all’ to ‘Applies completely’.”

	Does not apply at all				Applies completely
I understand advice from others to be an intrusion.	○	○	○	○	○
I become frustrated when I am unable to make free and independent choices.	○	○	○	○	○
Advice and recommendations usually induce me to do just the opposite.	○	○	○	○	○
I find contradicting others stimulating.	○	○	○	○	○
When something is prohibited, I usually think ‘that’s exactly what I am going to do’.	○	○	○	○	○

7. Explicitness of belief in the unhealthy = tasty intuition

“Please indicate how much you agree with the following statements.”

	1- I do not agree	2	3	4	5	6	7	8	9 - I agree a lot
Things that are good for me rarely taste good.	○	○	○	○	○	○	○	○	○
There is no way to make food healthier without sacrificing the taste.	○	○	○	○	○	○	○	○	○

8. General health interest

“Please indicate how much you agree with the following statements.”

	1- I do not agree	2	3	4	5	6	7 - I agree a lot
The healthiness of food has little impact on my food choices.	○	○	○	○	○	○	○
I am very particular about the healthiness of food I eat	○	○	○	○	○	○	○
I eat what I like and I do not worry much about the healthiness of food.	○	○	○	○	○	○	○
It is important for me that my diet is low in fat.	○	○	○	○	○	○	○
I always follow a healthy and balanced diet.	○	○	○	○	○	○	○
It is important for me that my daily diet contains a lot of vitamins and minerals.	○	○	○	○	○	○	○
The healthiness of snacks makes no difference to me.	○	○	○	○	○	○	○
I do not avoid foods, even if they may raise my cholesterol.	○	○	○	○	○	○	○

9. Dieting status and food allergies

“Are you currently on a diet?“

○ Yes

○ No

“Do you have any food allergies?”

○ Yes

○ No

10. Demographic questionnaire

“What is your gender?“

○ female

○ Male

○ Other: ___________

“What is your nationality?“ (Author note: multiple answers possible)

☐ Germany

☐ Austria

☐ Switzerland

☐ Other, EU

☐ Other, non-EU

“Is German you native language?“

- ○ Yes
- ○ No

“If German is not your native language, how well do you speak German?“

- ○ As well as my native language
- ○ Very well
- ○ Well
- ○ Moderately well
- ○ Not well
- ○ Very badly

“What year were you born in?”
Please indicate the year in 4 digits (e.g., 1990)

Birth year: ______

“What month were you born in?”

- ○ January
- ○ February
- ○ March
- ○ April
- ○ May
- ○ June
- ○ July
- ○ August
- ○ September
- ○ October
- ○ November
- ○ December

“How tall are you?”

Size in cm: ______

“What is your weight?”

Weight in kg: ______

“Are you currently employed?
Employment is understood as any occupation that is paid or associated with an income, regardless of its duration. Which on this list applies to you?”

- ○ I am full-time employed with 35 working hours or more per week

- ○ I am part-time employed with 15 to 34 working hours per week
- ○ I am part-time or hourly employed, with less than 15 working hours a week
- ○ I am on maternal/parental leave or other leave of absence
- ○ I am currently not employed.

“If you are not full-time or part-time employed: Which group on this list do you belong to?”

- ○ Student in a school of general education
- ○ Student (University or college)
- ○ Retiree, pensioner, in early retirement
- ○ Unemployed
- ○ Permanently unable to work
- ○ Housewife/househusband
- ○ Other

“What is the <u>highest</u> general education school leaving certificate that you have?”

- ○ I left school without graduation
- ○ I have a graduation of a compulsory school (E.g., lower secondary school or equivalent level of another school type)
- ○ I have a secondary school certificate or comparable graduation
- ○ I have a graduation from a vocational school or training
- ○ I have the general or subject-linked eligibility of university admission / A levels / High-school diploma
- ○ I have a university or college degree (Bachelor’s, Master’s, Diploma, licence, state examination, PhD, habilitation or other)
- ○ I have another type of certificate, namely: ____

11. Thanks and debriefing
